# Can minimally invasive transforaminal lumbar interbody fusion achieve a better clinical and radiological outcome than traditional open technique in isthmic spondylolisthesis?

**DOI:** 10.1186/s13018-024-04994-4

**Published:** 2024-08-29

**Authors:** Elsayed Mohamed Selim Ali, Amr Mohamed Eladawy, Tarek ElHewala

**Affiliations:** Orthopedic Department Faculty of Medicine, Zagazig University Hospital, Zagazig, Egypt

**Keywords:** Isthmic spondylolitheisis, MIS-TLIF, Minimal invasive, Spino-pelvic parameter

## Abstract

**Background:**

Spondylolisthesis is a prevalent condition in the lumbar spine that can cause low back pain, leg pain, neurogenic claudication, and impact health-related quality of life in symptomatic individuals.

**Aim:**

to assess the results of minimally invasive TLIF (MIS-TLIF) versus open-TLIF and the impact of correcting spino-pelvic parameters on the Health-Related Quality of Life (HRQoL) in patients with low-grade isthmic spondylolisthesis. The primary objective was to compare the effectiveness of both methods in correcting spinopelvic parameters. The secondary objectives involved comparing clinical improvement, operating time, blood loss, complications, and postoperative hospital stays between the two procedures.

**Patients and methods:**

Seventy-two patients with low-grade isthmic spondylolisthesis were enrolled in this retrospective cohort-control study, with a minimum follow-up period of 18 months. Disability was assessed using the Oswestry Disability Index (ODI), while back and leg discomfort were rated using the Visual Analogue Scale (VAS) for each patient. The measurements comprised the sacral slope (SS), pelvic tilt (PT), pelvic incidence (PI), and Meyerding slip grades. We measured lumbar lordosis (LL), and segmental lordosis.

**Results:**

The seventy-two patients were 60 female and 12 males. There was no statistically significant difference in the duration of operation between the two groups. In the MIS group, there was a notable reduction in blood loss, higher radiation exposure, and a shorter hospital stay (*P* < 0.001). The back VAS showed more favorable outcomes in the MIS-TLIF, while the leg VAS showed better results in the Open-TLIF in the early outcome. Both procedures significantly reduced leg and back pain VAS scores and ODI, with no notable difference between the two groups at the final follow-up. Post-surgery, the pelvic incidence (PI) and lumbar lordosis (LL) matched properly in all patients, showing a rise in LL and sacral slope along with a decrease in pelvic tilt.

**Conclusion:**

Both open-TLIF and MIS-TLIF are effective methods for correcting spino-pelvic parameters and improving HRQoL in patients with low-grade isthmic spondylolisthesis. The rapid improvement in back pain experienced by these patients favored the use of MIS-TLIF. However, the cost-effectiveness of this approach must be carefully evaluated.

**Supplementary Information:**

The online version contains supplementary material available at 10.1186/s13018-024-04994-4.

## Introduction

Spondylolisthesis is a ubiquitous lumbar spine disorder that often leads to symptoms such as neurogenic claudication, restricted function, and low back pain with or without leg discomfort. If conservative treatments fail to alleviate the symptoms, lumbar fusion surgery should be considered [1]. The goal of fusion surgery is to stabilize the spine and alleviate pain and neurological compression [2].

Transforaminal lumbar interbody fusion (TLIF) is the standard surgery for lumbar spondylolisthesis. It minimizes nerve root and thecal sac retraction while offering the benefits of overall fusion and maintaining or improving lumbar lordosis [1].

Minimally invasive spine (MIS) procedures yield equivalent or improved clinical and radiological outcomes compared to open treatments, along with decreasing soft tissue damage and its associated effects. MIS aims to decrease intraoperative blood loss, wound infections, postoperative hematomas, and maintain normal muscle function by preserving para-spinal muscular innervation. Additional advantages include accelerated wound healing, less analgesic requirement post-surgery, faster ambulation, and shorter hospitalization periods [3].

Drawbacks of MIS include extended operating times, heightened intraoperative radiation exposure due to prolonged and frequent fluoroscopy use, a challenging learning curve, and a potential rise in the risk of cage and pedicle screw misplacements and cage migrations [4].

Different research studied the relationship between pelvic incidence (PI), pelvic tilt (PT), and sacral slope with spinal deformities including spondylolisthesis, and their role in spinal sagittal alignment. Schwab F. et al. studied the relationships and variations of PI, PT, SS, LL, and thoracic kyphosis (TK) in a standard young adult group. They showed how these factors are interrelated and work together to support the overall balance of gravity over the femoral heads via muscle engagement. Recent findings suggest that a high Pelvic Incidence (PI) may be associated with adult individuals who have low-grade L5-S1 spondylolisthesis [5].

The Spinal Deformity Study Group (SDSG) introduced a classification system that relies on radiographic assessment of slip grade and spino-pelvic alignment, including pelvic incidence (PI), sacro-pelvic alignment, and spinal balance. Most guidelines and studies on spondylolisthesis focus on slip grade, however some research also highlights the significance of sacro-pelvic morphology and spino-pelvic alignment in assessing and treating spondylolisthesis [6].

In order to better understand how correcting spino-pelvic parameters impacts the health-related quality of life (HRQoL) of individuals with low-grade spondylolisthesis, we conducted this study to compare the results of open and minimally invasive TLIF surgeries. A comprehensive analysis of the effects of these treatment modalities for low-grade spondylolisthesis was our goal.

### Patients and methods

A retrospective cohort-control study was conducted to compare the effectiveness of Minimally Invasive Transforaminal Lumbar Interbody Fusion (MIS-TLIF) with Open Transforaminal Lumbar Interbody Fusion (Open-TLIF) in treating low-grade isthmic spondylolisthesis, which was carried out at the orthopedic department’s spine unit at our university hospital from December 2017 to December 2020 with a minimum 18-month follow-up duration, including 72 patients. We postulate that, in comparison to conventional open TLIF, MIS-TLIF will provide better radiological and clinical results. Our primary goal was to evaluate the corrective power of the two methods according to the spino-pelvic parameters. We aimed to assess clinical improvement, operating time, blood loss, complications, and postoperative hospital stays between the two procedures as our secondary objective.

The trial participants had low-grade isthmic spondylolisthesis, axial low back pain, and/or leg discomfort that persisted despite undergoing medical treatment including rest, non-steroidal anti-inflammatory drugs, muscle relaxants, physiotherapy, and lumbosacral support for at least six months. High-grade spondylolisthesis, severe osteoporosis, previous spinal surgery, spinal tumor, trauma, and infections were all excluded as contributing factors. Before the surgery, every patient provided their informed consent, and none of them were lost throughout the follow-up period.

Each patient had clinical evaluation before the surgery using the Oswestry Disability Index (ODI) to evaluate disability and the Visual Analogue Scale (VAS) to quantify leg and back pain. Demographic data, including age, sex, occupation, smoking status, and Body Mass Index (BMI), was collected from all patients.

Anteroposterior, lateral, and dynamic lumbar spine standing X-rays were used to evaluate each patient’s radiological status before surgery. An anterior–posterior and lateral X-ray from the base of the head to the tailbone was conducted. The radiographic analysis software Surgimap Spine (Nemaris Inc, New York, NY) was used to measure spino-pelvic parameters. Measurements were recorded for pelvic tilt (PT), pelvic incidence (PI), sacral slope (SS), and Meyerding slip grades. We assessed the segmental lordosis of the affected segment by measuring the angle between the upper endplate of the slid vertebra and the top endplate of the lower one, as well as lumbar lordosis (LL). All measures were taken by two experienced spine surgeons and then repeated by the same surgeons a month later. When they took the second round of measurements, the examiners were blind to their own previous measurements. The final analyses were performed using the average values of the measurements obtained at two separate time points. All patients got pre-operative MRI scans which consisted of sagittal, coronal, and axial views.

**Fig. 1:**
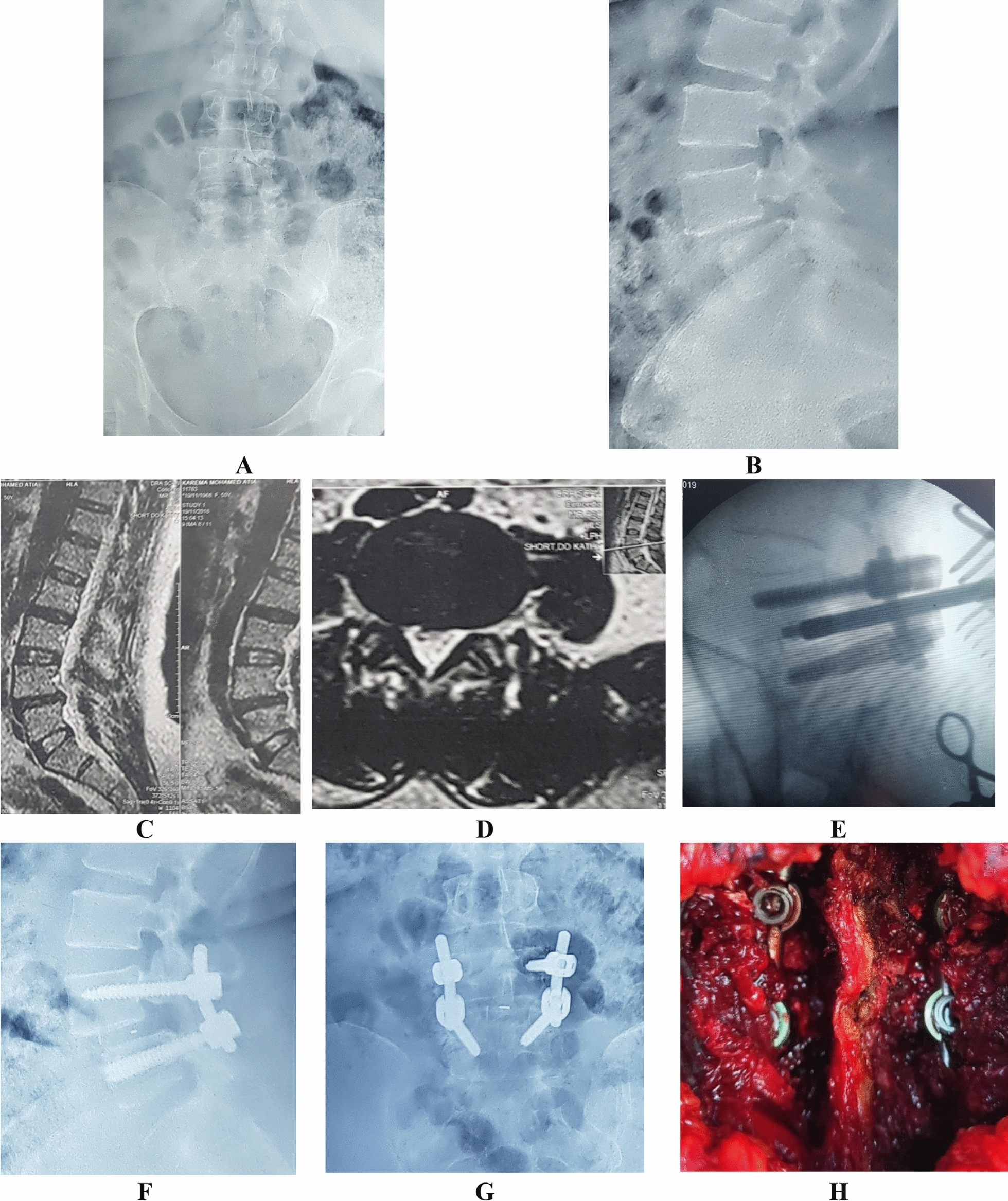
The open TLIF case, **A** and **B** anterior–posterior and lateral radiology of the slipped level, **C** and **D** MRI sagittal and axial cut, **E** intraoperative photo during insertion of the cage, **F** and **G** the final Anterior–posterior and lateral view of the operated level, and finally **H**: intra-operative photo of the operative field

**Fig. 2:**
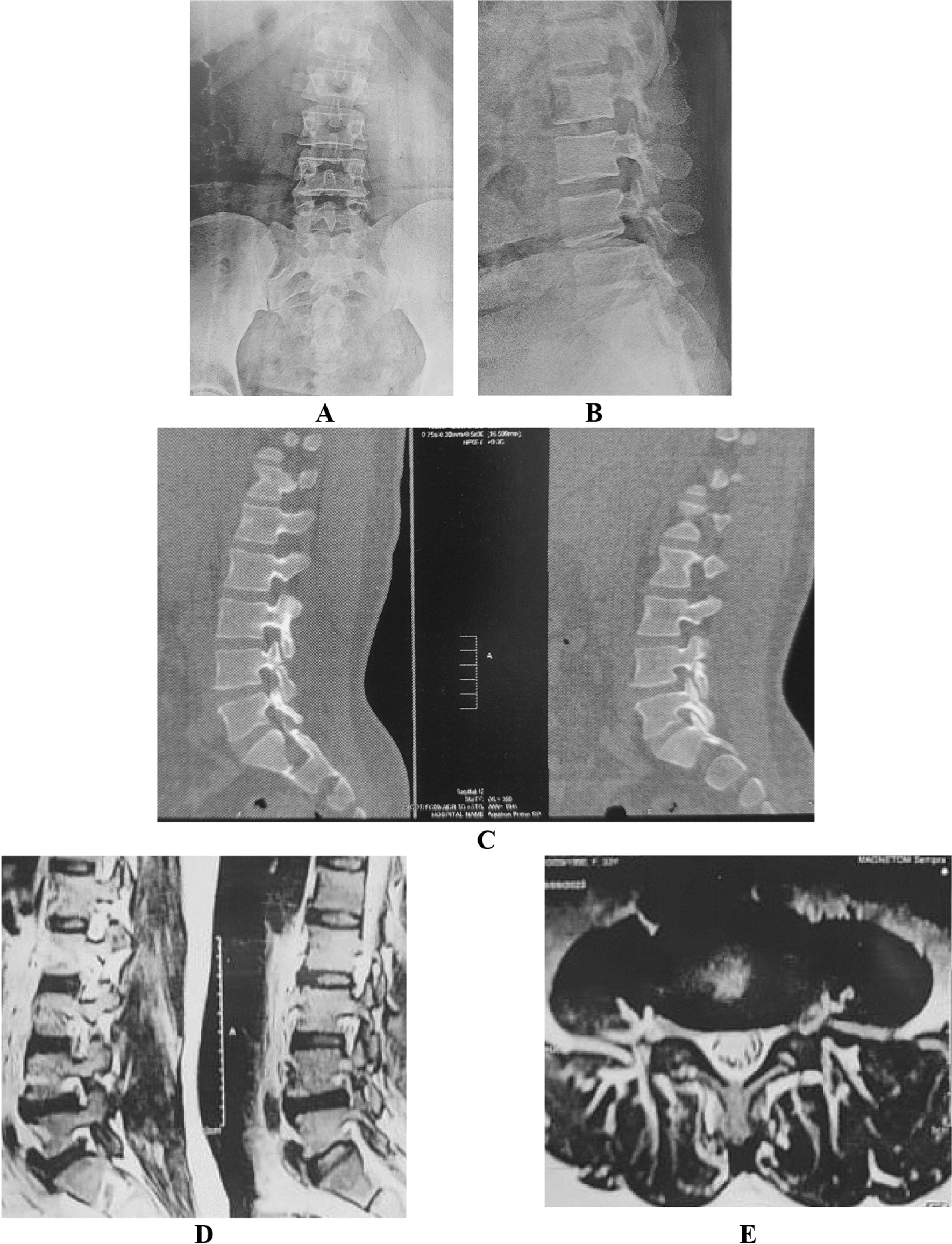
The MIS-TLIF case, **A** and **B** anterior–posterior and lateral radiology of the slipped level, **C** CT scan of the fractured pars, **D** and** E** showing MRI sagittal and axial cut of the slipped level

**Fig. 3:**
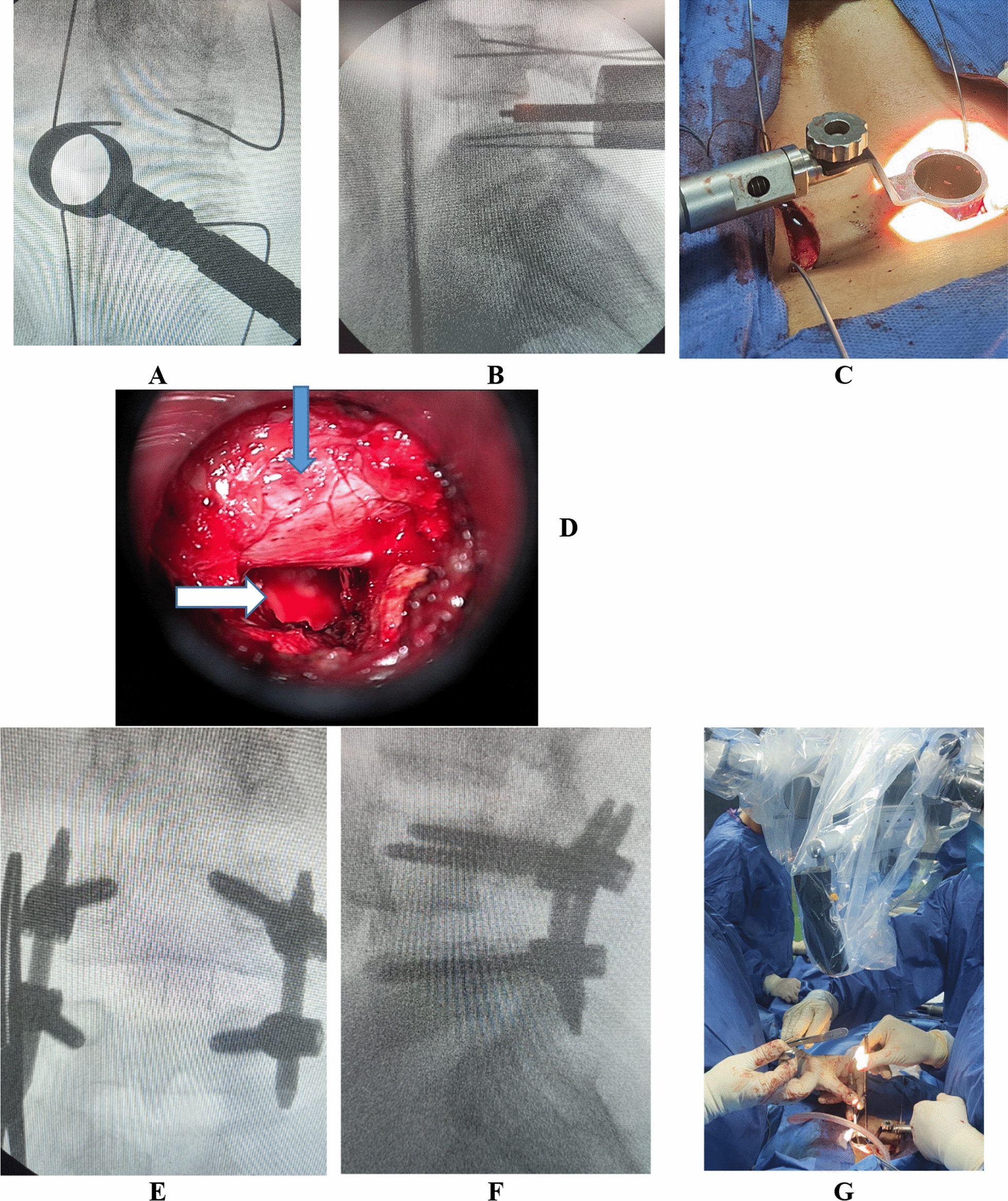
operative photos of the MIS-TLIF case: **A** showing the guide wires placed in the pedicles of the L4-5 with the tube system fixed over the facet joint before the osteotomy, **B** lateral radiology view during insertion of the cage, **C** intraoperative photo with the tube system in place with the percutaneous guide wires in place, **D** intraoperative microscopic picture of the dura (blue arrow) and the window for the insertion of the cage marked by the white arrow, **E** and **F** the final anterior–posterior and lateral view of the slipped level after insertion of the rods and **G** intraoperative photo of the operative microscope used

TLIF was used to treat the 72 participants with low grade lytic spondylolisthesis. They were divided into two groups: group A, comprising 40 patients who underwent open-TLIF, and group B, comprising 32 patients who underwent MIS-TLIF. Both groups used PEEK cages filled with autologous bone graft (taken from the laminectomy and the facets) and included posterior spinal fixation with pedicular screws using either an open or percutaneous approach. The open approach technique employed in this investigation was comparable to the methodology utilized in a previous study[7] (Fig. 1). Similarly, the MIS approach in this study was based on the methodology outlined in this other work [8], with same steps followed (Figs. 2 and 3). All patients in the study were operated by the same surgical team.

### Follow-up

Patients were encouraged to ambulate as early as the first postoperative day. Plain x-ray was ordered for the patients on day two postoperative. The patients were advised to attend the out-patient clinic in the first two weeks then regularly every three months.

### Statistical analysis

A sample size was calculated using openepi.com [9], with the assumption that the MIS-TLIF would make a 30% increase in the segmental lordosis in comparison to the open TLIF, depending on our previous study [8], with the assumption that the open TLIF group is the standard technique so it will be larger in size. The calculated sample size ranged from 70 to 85 patients. We approximated calculated numbers for groups to (40) patients in group A and (32) patients in group B, according to Kelsey and Fleiss methods (supplementary figure). Continuous variables were expressed as the Mean ± SD (Range). The categorical variables were expressed as a number (percentage). Data found to be normally distributed were analyzed using the independent sample t-test, while Repeated Measures ANOVA was used for analysis of repeated measured data within variables. Non-parametric data were analyzed using the Mann Whiteny U-test for rank sum of independent samples and Wilcoxon test measured if there was difference in dependent groups All statistical analyses were performed with SPSS version 24.0 for windows (SPSS Inc., Chicago, IL, USA), where *P* < 0.05 was considered statistically significant.

## Results

### Patients demographics

This study included 72 patients, 60 of whom were female and 12 of whom were male, with single-level low-grade isthmic spondylolisthesis. Table 1 shows that there were no significant differences between the two groups in terms of patient demographics. The TLIF procedure was performed openly in 40 patients (55.5%), and minimally invasively in 32 individuals (44.4%).

**Table 1:** Demographic data of the included patients in the study

Demographic data	Open-TLIF (n = 40)	MIS-TLIF (n = 32)	Test	*p*-value
Age	44.80 ± 4.519	44.47 ± 5.168	Z = − 0.410	*p* = 0.682
Sex				
Male	6 (15%)	7 (21.9%)	Z = − 1.058	*p* = 0.290
Female	34 (85%)	25 (78.1%)		
Main complain				
LBP	11 (27.5%)	8 (25.0%)	Z = − 0.237	*p* = 0.812
LBP-LLP	29 (72.5%)	24 (75.0%)		
Levels				
L5-S1	25 (62.5%)	18 (56.3%)	Z = − 0.534	*p* = 0.594
L4-L5	15 (37.5%)	14 (43.8%)		
Preoperative slip%	22.38 ± 1.944	22.59 ± 1.643	Z = − 0.259	*p* = 0.795
Follow-up period	23.75 ± 2.010	23.69 ± 1.281	*t* = 0.160	*p* = 0.873

### Operative data

The length of surgery differed statistically significantly between the two groups, with open procedures having a lower operative time. Furthermore, the open approach had a significantly lower radiation exposure than the MIS opens. In comparison to the MIS-TLIF group, the statistical analysis demonstrated a statistically significant decrease in both intraoperative and postoperative blood loss in the MIS group, with a considerably shorter hospital stay (*p* < 0.001) (Table 2).

**Table 2:** Operative data

Operative data	Open-TLIF (n = 40)	MIS-TLIF (n = 32)	Test	*p*-value
Operative time (in min)	114.40 ± 8.057	120.94 ± 6.891	Z = − 3.354	*p* < 0.001
Intraoperative blood loss (in ml)	538.75 ± 50.94	245.31 ± 34.73	*t* = 28.970	*p* < 0.001
Radiation exposure (in sec)	20.13 ± 6.276	60.25 ± 7.030	*t* = − 25.230	*p* < 0.001
Hospital stay (in day)	2.55 ± 0.504	1.47 ± 0.567	Z = − 5.995	*p* < 0.001

### Pain and functional outcomes

#### Modified Oswestry disability index (Fig. 4)

**Fig. 4:**
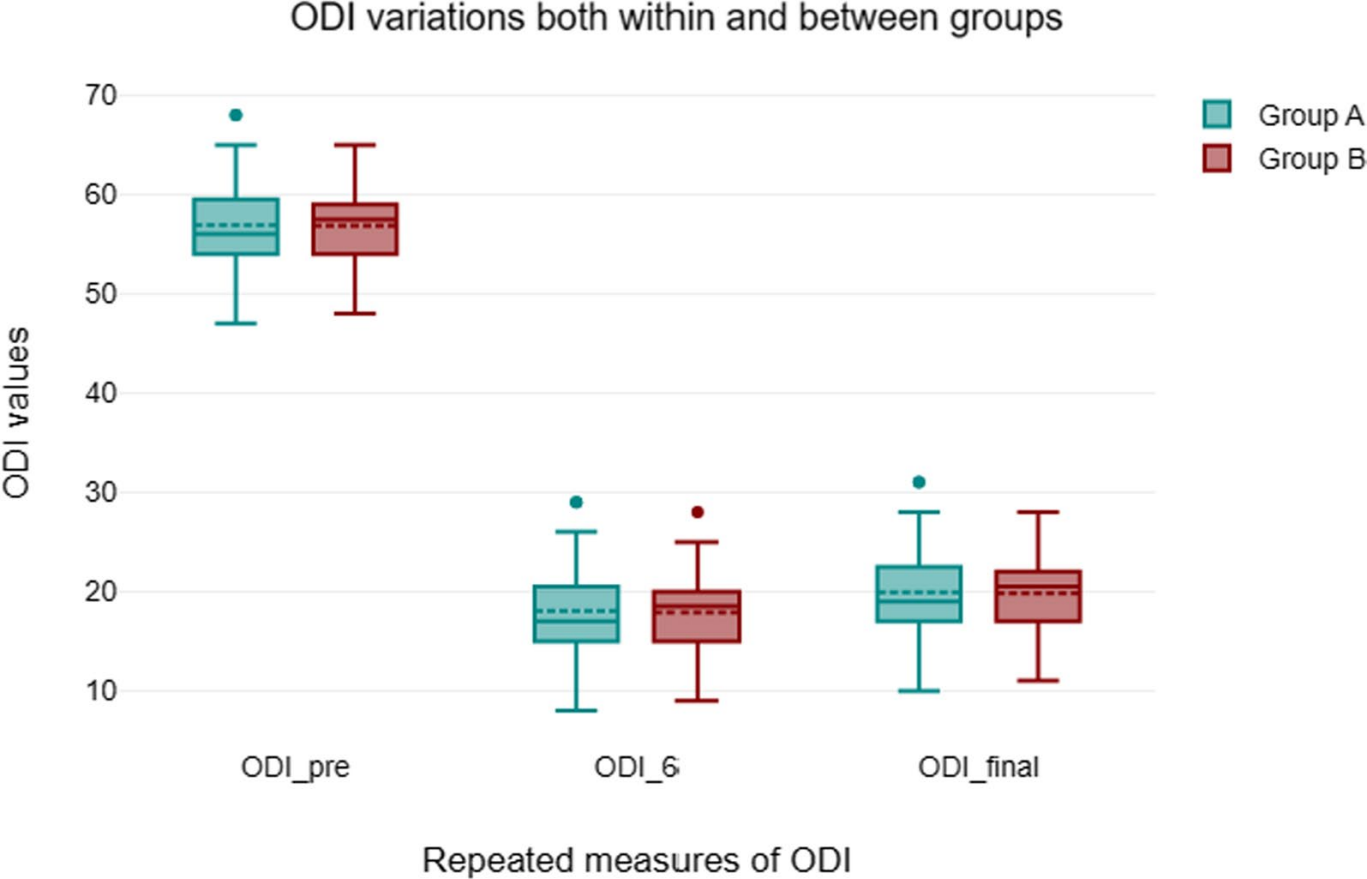
Modified Oswestry disability index

The Oswestry disability score was used to compare Open-TLIF with MIS-TLIF before, after surgery, and at final follow-up. No statistically significant difference was found between the two groups. Within each group, however, there was a discernible change from pre- to post-operative ODI values that persisted through the last follow-up, and this change was highly statistically significant.

#### Visual analogue scale back and leg pain (Figs. 5, 6)

**Fig. 5:**
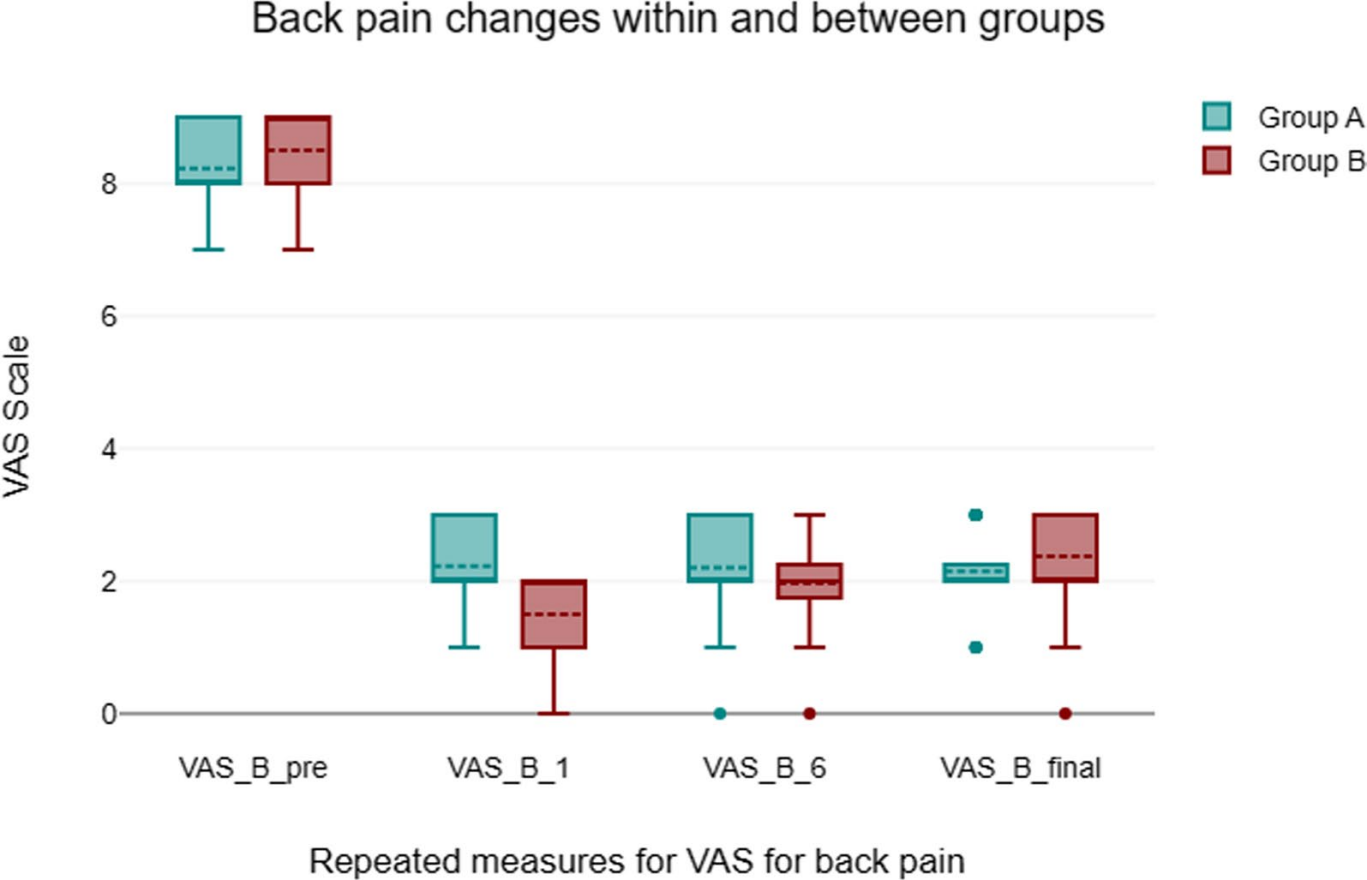
VAS for back pain

**Fig. 6:**
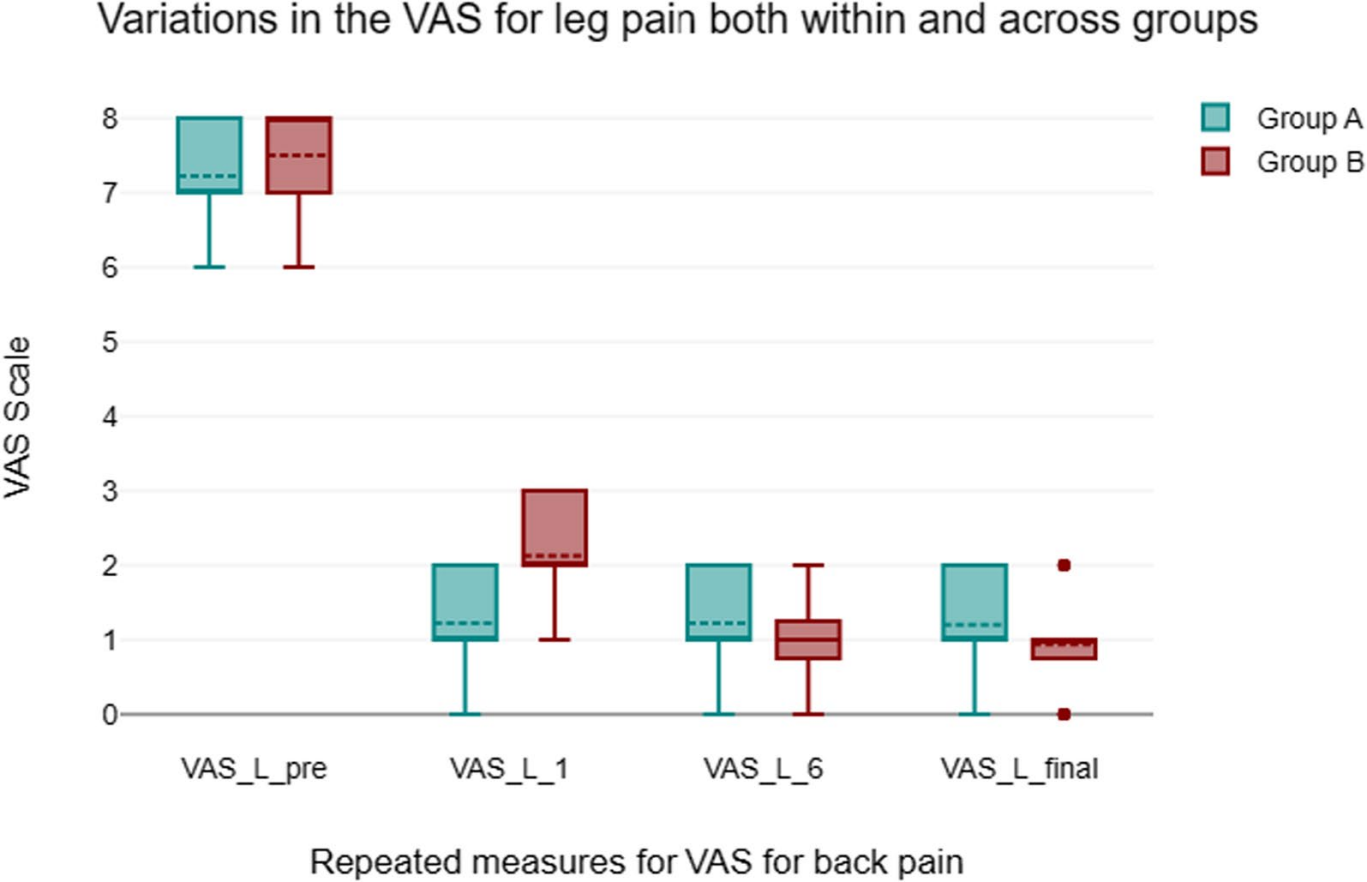
VAS for leg pain

Each group’s back and leg pain were assessed using a VAS score. The results demonstrated a marked improvement in back pain scores across all groups from preoperative to postoperative data till the final follow-up. VAS score for Back pain improved from preoperative with the MIS-TLIF group outperforming the open group in the first month. When comparing the groups at pre- and post-operative follow-up, there was a statistically significant difference between the open and MIS-TLIF groups on the VAS for leg pain within the first month in favor the open group.

#### Radiological parameters including the spino-pelvic parameters (Table 3)

**Table 3:** Radiological outcome

Radiographic data		Open-TLIF (n = 40)	MIS-TLIF (n = 32)	Test	*p*-value
Lumbar lordosis	Pre	71.50 ± 4.472	72.38 ± 3.687	*t* = − 0.910	*p* = 0.366
	Final	63.23 ± 5.206	69.13 ± 3.230	*t* = − 5.889	*p* < 0.001
Test (*p*-value)		*t* = 14.278 (*p* < 0.001)	*t* = 7.851 (*p* < 0.001)		
Segmental lordosis	Pre	0.58 ± 1.394	0.63 ± 1.385	Z = − 0.273	*p* = 0.785
	Final	− 6.23 ± 2.684	− 3.88 ± 2.240	*t* = − 4.049	*p* < 0.001
Test (*p*-value)		Z = − 5.523 (*p* < 0.001)	Z = − 4.958 (*p* < 0.001)		
Pelvic incidence	Pre	53.85 ± 5.082	55.44 ± 4.064	*t* = − 1.473	*p* = 0.145
	Final	53.83 ± 5.002	55.47 ± 3.951	*t* = − 1.558	*P* = 0.124
Test (*p*-value)		*t* = 0.298 (*p* = 0.767)	*t* = − 0.571 (*p* = 0.572)		
Sacral slope	Pre	34.48 ± 5.435	37.88 ± 4.412	*t* = − 2.930	*p* = 0.005
	Final	35.88 ± 5.080	38.88 ± 4.148	*t* = − 2.758	*P* = 0.007
Test (*p*-value)		*t* = − 4.350 (*p* < 0.001)	*t* = − 4.209 (*p* < 0.001)		
Pelvic tilt	Pre	19.38 ± 2.404	17.56 ± 3.015	*t* = 2.768	*p* = 0.008
	Final	17.95 ± 2.364	16.59 ± 2.838	*t* = 2.168	*p* = 0.034
Test (*p*-value)		Z = − 3.771 (*p* < 0.001)	Z = − 3.372 (*p* = 0.001)		
PI-LL mismatch	Pre	17.65 ± 3.549	18.38 ± 3.396	*t* = − 1.129	*p* = 0.259
	Final	9.40 ± 1.865	9.47 ± 1.831	*t* = − 0.156	*p* = 0.876
Test (*p*-value)		Z = − 5.520 (*p* < 0.001)	Z = − 4.945 (*p* < 0.001)		
Slip percentage	Pre	22.38 ± 2.404	22.59 ± 3.015	*t* = − 0.259	*p* = 0.795
	Final	5.45 ± 1.600	7.88 ± 1.129	*t* = − 5.550	*p* < 0.001
Test (*p*-value)		Z = − 5.526 (*p* < 0.001)	Z = − 4.959 (*p* < 0.001)		
Disc height (in cm)	Pre	1.006 ± 0.146	1.023 ± 0.097	Z = − 0.863	*p* = 0.388
	Final	1.667 ± 0.189	1.746 ± 0.177	Z = − 1.755	*p* = 0.079
Test (*p*-value)		Z = − 5.516 (*p* < 0.001)	Z = − 4.940 (*p* < 0.001)		

**Fig. 7:**
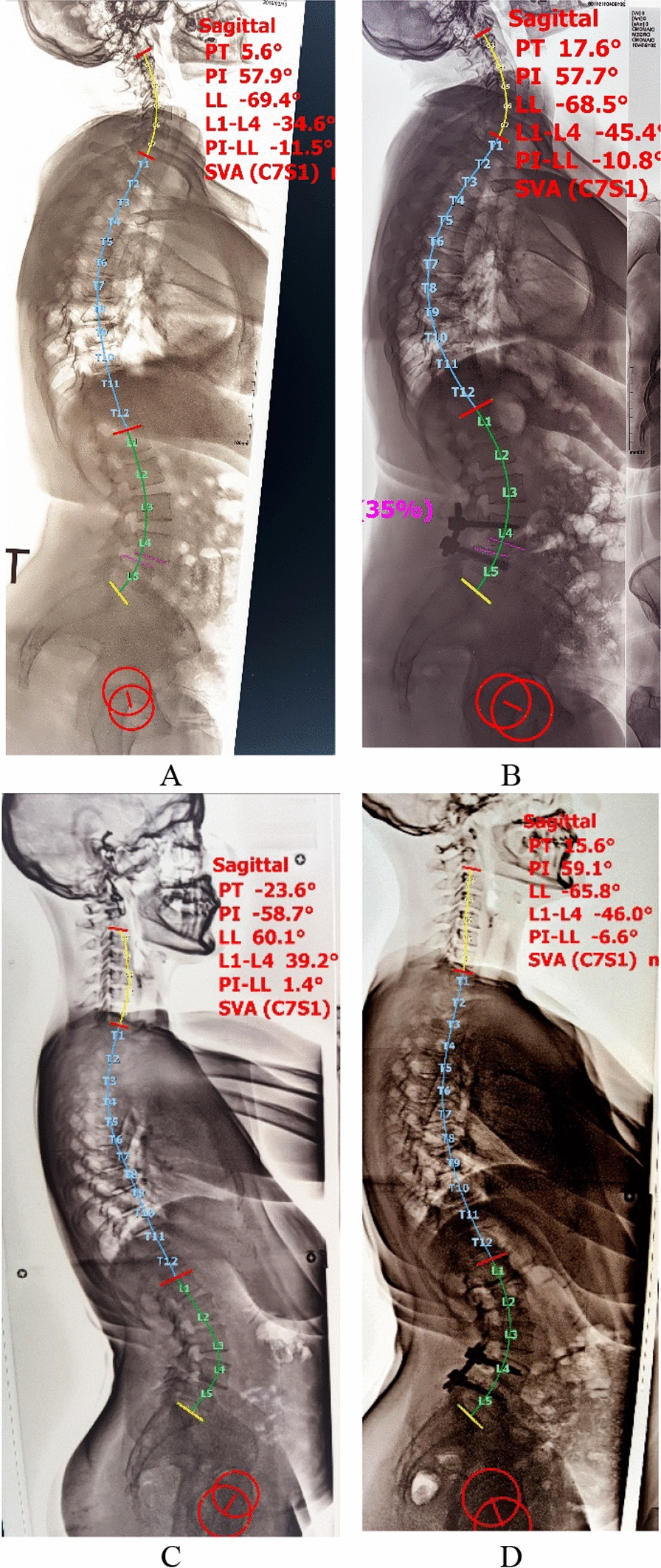
Long standing radiographyof the whole spine pre and postoperative of the open-TLIF case in (**A** and **B**) and the MIS-TLIF case (**C** and **D**) with measurement of the spinopelvic parameters

Between the preoperative and final follow-ups, both groups had a significant reduction in slip % and disc height correction, as well as a significant improvement in lumbar and segmental lordosis in the affected level. In addition, the sacral slope and pelvic tilt revealed considerable degrees of correction, which correlated with the correction of the pelvic incidence lumbar lordosis mismatch. For data analysis between groups, there was a substantial difference in parameter correction, with the open technique having the most correction values when considering LL, SL, slip percentage, and disc height adjustment (Fig. 7).

#### The correlation between the sagittal parameters and the functional outcome at final follow up (Table 4)

**Table 4:** The result of the pearson correlation between the sagittal parameters and the functional outcome at final follow up

	ODI final		VAS Leg pain final		VAS back pain final	
	r	*p* value	r	*p* value	r	*p* value
Both groups						
LL	0.14	0.242	− 0.32	0.006	− 0.09	0.444
PI-LL MIS	0.12	0.326	− 0.03	0.804	− 0.13	0.291
PT	0.03	0.786	− 0.02	0.89	− 0.14	0.257
Disc height	− 0.29	0.014	− 0.18	0.126	− 0.1	0.404
Slip%	0.09	0.456	− 0.12	0.332	0.04	0.712
Seg lord	0.07	0.574	− 0.18	0.121	− 0.01	0.908
MIS group						
LL	0.3	0.096	− 0.03	0.887	0.02	0.908
PI-LL MIS	0.01	0.953	− 0.03	0.879	− 0.02	0.933
PT	− 0.05	0.794	0.16	0.394	0.19	0.295
Disc height	− 0.45	0.01	− 0.22	0.229	− 0.35	0.05
Slip%	0.08	0.671	0.33	0.064	0.1	0.582
Seg lord	0.34	0.054	− 0.25	0.162	− 0.13	0.47
Open group						
LL	0.13	0.427	− 0.39	0.012	− 0.4	0.01
PI-LL MIS	0.18	0.263	− 0.03	0.876	− 0.1	0.521
PT	0.09	0.585	− 0.29	0.065	− 0.16	0.315
Disc height	− 0.21	0.195	− 0.09	0.574	− 0.02	0.881
Slip%	0.15	0.37	− 0.16	0.315	− 0.1	0.53
Seg lord	− 0.05	0.751	− 0.02	0.914	− 0.08	0.638

All the underlined data are significant as p value less than 0.05 is significant and p value less than 0.001 is highly significant

During the study of the results of the correlation (Pearson) between the sagittal parameters and the functional outcome (VAS and ODI) for both groups (open and MIS), there was no statistically significant correlation discovered except for the a negative correlation with lumbar lordosis and VAS_L (r (70) =  − 0.3, *p* = 0.006.) and between the disc height and the ODI (r (70) =  − 0.2, *p* = 0.01).

In the MIS group, the result of the Pearson correlation showed that there was a statistically significant negative correlation between ODI final and Disc height final (r(30) =  − 0.4, *p* = 0.01) and a statistically significant negative correlation between VAS Back and Disc height r(30) =  − 0.35, *p* = 0.05).

In the open group the result of the Pearson correlation showed that there was a statistically significant negative correlation between LL and VAS Leg (r (38) =  − 0.3, *p* = 0.01) and a statistically significant negative correlation between LL and VAS Back (r (38) =  − 0.4, *p* = 0.01).

### Complications

One patient in the open group (2.5%) had a superficial wound infection five days following surgery. Once the first week had passed, the infection was under control thanks to intravenous antibiotics and frequent dressing changes. There were five cases (6.9%) of accidental durotomy: two in the open (5%) setting and three in the MIS-TLIF (9.4%) setting. Patients with MIS-TLIF had a muscle graft in surgicel with a tight fascia closure, whereas those with open lesions were treated by direct dural suture. Only one case from the MIS-TLIF group required revision with microscopic dural repair with durotomy suturing; all others did not require revision. Two patients in the MIS-TLIF (6.3%) groups had screws that were not in proper places, but no additional intervention was necessary in these circumstances. In neither group did any neurological complications occur.

## Discussion

There are several surgical procedures available for the treatment of lytic spondylolisthesis, such as open and minimally invasive transforaminal lumbar interbody fusion (TLIF). A study has indicated that a combination of unilateral TLIF and pedicle screw fixation can effectively treat low-grade spondylolisthesis. This approach offers the potential advantages of minimally invasive percutaneous long-arm pedicle screws in spondylolisthesis surgery. According to the study conducted by Nooraie et al., there was no statistically significant disparity observed in the outcomes of low-grade lytic spondylolisthesis when comparing spinal decompression, stabilization, and fusion, as well as stabilization and fusion without decompressive laminectomy[10].

The MIS-TLIF technique involves the utilization of a para-median skin incision, muscle dilation, or splitting to gain access to the posterior lumbar interbody space. To maintain an open access channel, various mechanisms such as sleeves, tubes, or cylindrical retractor blades are employed [11]. To mitigate the dependence on the indirect decompression technique with a unilateral approach, bilateral decompression was employed in the present investigation. Consequently, there were no instances of contralateral radiculopathy observed in any of our patients during the follow-up period.

The development of minimally invasive technologies for spinal surgery over the last few decades has undoubtedly resulted in the transition from Open-TLIF to MIS-TLIF. The MIS-TLIF technique has gained popularity for its advantages over open-TLIF, such as a smaller incision, less bleeding, and faster recovery. Minimally invasive spine surgery strives to achieve the same results as open procedures but in a less stressful manner [7].The purpose of this study was to evaluate and compare the safety and efficacy of Open-TLIF and MIS-TLIF in the treatment of low-grade, single-level isthmic spondylolisthesis.

The primary concern expressed by our patients encountered pain in the lower back together with radiating pain in the lower limbs. This was reported by 72.5% of patients who underwent open-TLIF and 75% of patients who underwent MIS-TLIF. The most frequently operated level was L5-S1. In our study group, it was evident that female patients had a greater prevalence of lytic spondylolysis compared to male patients. This was observed in both the open-TLIF group (85%) and the MIS-TLIF group (78.1%). However, there was no significant difference between the two groups in terms of demographic data.

Regarding the surgical data, it came to light that the open TLIF procedure had favorable outcomes in terms of operative time and radiation exposure. Fluoroscopy-based minimally invasive surgical (MIS) techniques expose patients and surgical workers to significant doses of radiation, as it is necessary for determining the anatomical location. Multiple studies have shown that the use of fluoroscopy is more extensive in the treatment of lumbar issues with MIS-TLIF compared to Open-TLIF [8, 12, 13]. The current analysis found that the radiation exposure (measured in seconds) was much lower in the open TLIF compared to the MIS-TLIF (20.1 ± 6.3 vs 60.3 ± 7, respectively), indicating a preference for the open TLIF procedure. Furthermore, we found that the duration needed to insert the percutaneous screw and guide wire was the primary factor contributing to the fluoroscopy time.

In the current study, the operating time in the MIS group was longer. This could be likely additional time was spent assembling the tubular retractors and accurately setting the screws using fluoroscopy. Specifically, the operating time for Open-TLIF was 114.4 ± 8 min, whereas for MIS-TLIF it was 120.9 ± 6.9 min. The visual field during minimally invasive surgery is narrower compared to standard surgery. The surgeon must possess significant practical expertise and a comprehensive comprehension of anatomy [14]. Multiple studies have demonstrated that the MIS-TLIF technique outperformed Open-TLIF in terms of intraoperative bleeding, postoperative drainage, and duration of hospitalization [3]. Our study’s results were in line with their conclusions on the smaller incisions, reduced tissue injury, and the utilization of a tubular retractor.

Because many of the possible advantages of MIS-TLIF may manifest themselves in the early post-operative recovery phase, favoring MIS-TLIF, early results in open-TLIF versus MIS-TLIF comparisons are significant [14]. When comparing MIS-TLIF to open-TLIF for VAS back pain, the results were clear. The open approach may result in more severe muscle injury and more muscle strapping compared to MIS-TLIF, which may explain why the former causes more discomfort in the first month after surgery (2.2 ± 0.7 vs 1.2 ± 0.7, respectively). The VAS back pain has a short-term effect, but there was no significant difference between the two groups at the final follow-up in the long-term follow-up (Open TLIF 2.1 ± 0.5 vs MIS-TLIF 2.4 ± 0.7). In terms of early VAS back pain, Xie et al. [15] discovered significant differences between the MI-TLIF and Open-TLIF groups (MD =  − 1; 95%CI =  − 1.98, − 0.2; *p* = 0.02). Moreover, research was limited to the early groups experiencing VAS back discomfort (I2 = 90%, *P* < 0.001) [16]. There is no discernible change in VAS scores for back pain in late follow-up trials [1, 6, 8].

Opposite to MIS-TLIF, Open TLIF showed a notable reduction in VAS leg pain in the initial one month following surgery (1.5 ± 0.6 vs 2.1 ± 0.7, respectively). This could be because surgeons are more comfortable with the open standard method, there is less manipulation of neural tissues and retraction, disc height restoration is successful, and nerve root and dura release are well-executed. In the last follow-up, however, there was no significant difference between the two methods with respect to radiculopathy (Open TLIF 1.2 ± 0.6 vs MIS-TLIF 0.9 ± 0.7).

Consistent with the increase in patient functional outcome indicated by ODI, both groups saw an improvement in back and leg discomfort. Both groups’ ODIs were significantly adjusted between the preoperative and final follow-up periods, with the Open group showing a difference of 56.9 ± 5.1 vs 19.9 ± 5 and the MIS-TLIF group showing a difference of 56.8 ± 3.8 vs 19.8 ± 3.8. The results show that in cases of single level spondylolisthesis, the MIS-TLIF can improve life function just as much as the traditional open-TLIF. Despite the brief duration of this study’s follow-up, the ODI scores were comparable to those of other research that compared open-TLIF and MIS-TLIF in the treatment of single-level spondylolisthesis and found that both methods improved ODI scores [8, 16, 17].

Schwab et al. demonstrate that the sagittal plane is the main driver of disability in patients with ASD and indicate that among the sagittal radio-graphical parameters, SVA, PT, and PI-LL mismatch are the key factors that impact disability. They have proposed threshold values of sagittal spino-pelvic alignment that should be achieved with spinal reconstructive procedures to obtain satisfactory outcomes in terms of HRQOL. Using these parameters, it is possible to predict theoretical values of regional sagittal parameters [5, 18]. More recently, Shimokawa et al. [19] have reported high correlations between pelvic retroversion (measured by the PT) and sagittal vertical axis (SVA) with HRQOL scores*.* Although pelvic retroversion may compensate for sagittal balance, it significantly lowers quality of life. In addition to SVA, PT should be taken into consideration to enhance the assessment of patients with lumbar problems [19].

Spondylolisthesis is characterized by three basic abnormalities: segmental kyphosis, disc height decrease, and vertebral slippage. When forward slippage happens, the lumbar lordosis diminishes due of the disc degeneration and the segmental kyphosis generated by the slippage. In order to compensate the pelvic retroversion-induced displacement, the upper spinal segments hyperextend [8, 20]. Although this compensation improves SVA, patients who have a high PT and an ongoing PI-LL mismatch may be at risk for severe impairment. Additionally, it is believed that PT couldn’t go above 20–22°[21].

We evaluated the PI-LL mismatch in addition to the alterations and corrections to various lumbo-pelvic parameters, including the SS, PT, LL, and PI, in relation to the sagittal parameters that needed adjustment. Additionally, we investigated at how the spondylolisthesis’s slip percentage changed, as well as how the disc angle (segmental lordosis) and disc height changed. There was no change in PI values between preoperative and final follow-up for either the open TLIF group (*p* = 0.7) or the MIS-TLIF group (*p* = 0.5). This lends credence to the theory that the PI remains constant across all individuals.

When comparing the two groups’ pre- and post-operative follow-ups on the remaining parameters, we find that the open and MIS groups differ significantly, especially in the post-operative final follow-up. In comparison to the MIS group, the open group achieved greater corrections for LL, SS, PT, PI-LL mismatch, and segmental lordosis. Possible causes include the simplicity of using a lordotic rod, applying compression on the screws, and doing away with facet joints entirely. Both groups displayed a clear restoration of height on the disc, with no discernible difference between them. One possible explanation is that the transforaminal corridor allowed for the application of a big cage to both groups.

Reduction of the slip percentage can increase the area of intervertebral bone grafting and return the spine to its physiological position with restoration of the sagittal balance [22]. It is unclear if forceful reduction is necessary during surgery for low-grade Isthmic spondylolisthesis [23, 24]. When comparing each group’s preoperative status with the results of the last follow-up, a substantial improvement in the vertebral slip percentage was seen in our study (*P* < 0.001). Furthermore, at final follow-up time point, there was statistically significant difference in the vertebral slip ratio between the MIS-TLIF group and the Open-TLIF group (*P* < 0.001). It demonstrated that both groups had produced positive reduction outcomes with significant improvement in the open group at the final follow-up.

The most frequent complication (6.9%) seen in this study was accidental durotomy, which typically occurred during the placement of the TLIF cage. A single instance within the MIS group necessitated microscopic repair revision. There were claims that the fluoroscopic guided placement of pedicle screws during MIS operations contributed to the screws being misplaced. The accuracy of fluoroscopy-guided percutaneous pedicle screw placement in minimally invasive TLIF was recently investigated by El-Desouky et al. [25]. The researchers found that the technique was safe, with a total incidence of 13.9% of pedicles’ wall violations and 0.48% of patients reporting complaints due to these screws that were placed incorrectly. Since two patients (6.2% of the total) in the MIS group did not experience any discomfort because of the screws that were implanted, no additional action was necessary.

Neither group experienced a serious infection throughout the current investigation. The Open-TLIF group had a single case of superficial incision infection (2.5%). This was less than the overall prevalence of surgical site infection which was calculated to be (4.2%) in the MENA region [26]. The patient’s full recovery was achieved using antibiotics and frequent dressing changes. Our patients did not have any neurological complications. At the final follow-up, all cases in both research groups showed full interbody fusion with no evidence of cage displacement or screw loosening.

## Conclusion

Both open-TLIF and MIS-TLIF are effective methods for correcting spino-pelvic parameters and improving the health-related quality of life in patients with low-grade isthmic spondylolisthesis. The rapid improvement in back pain experienced by these patients favored the use of MIS-TLIF. However, the cost-effectiveness of this approach must be carefully evaluated.

Patients with low-grade isthmic spondylolisthesis can benefit from both open-TLIF and MIS-TLIF, two dependable procedures for correcting spino-pelvic parameters and improving their health-related quality of life. Although MIS-TLIF had the advantage because to the patients’ quick recovery from back pain, the cost-effectiveness of this must likely be considered.

## Limitations

First short term of follow up together with being a retrospective study, Second, the sample sizes in each arm of the trial were small, which could have an impact on the findings; third, a correlation analysis between the clinical effect and radiological evaluation was not done; and fourth, no data was gathered regarding the height of the intervertebral foramen. We recommend long-term follow up to detect more changes in results with multicenter study with wide base of population and large number of cases.

### Supplementary Information

Supplementary file 1. 

## Data Availability

All data is available in the main text or the supplementary material.
