# Supplementary figures and images for: Can minimally invasive transforaminal lumbar interbody fusion achieve a better clinical and radiological outcome than traditional open technique in isthmic spondylolisthesis?
Source: J Orthop Surg Res. 2024 Aug 29;19:523. doi: 10.1186/s13018-024-04994-4 (PMC11360516; doi:10.1186/s13018-024-04994-4)

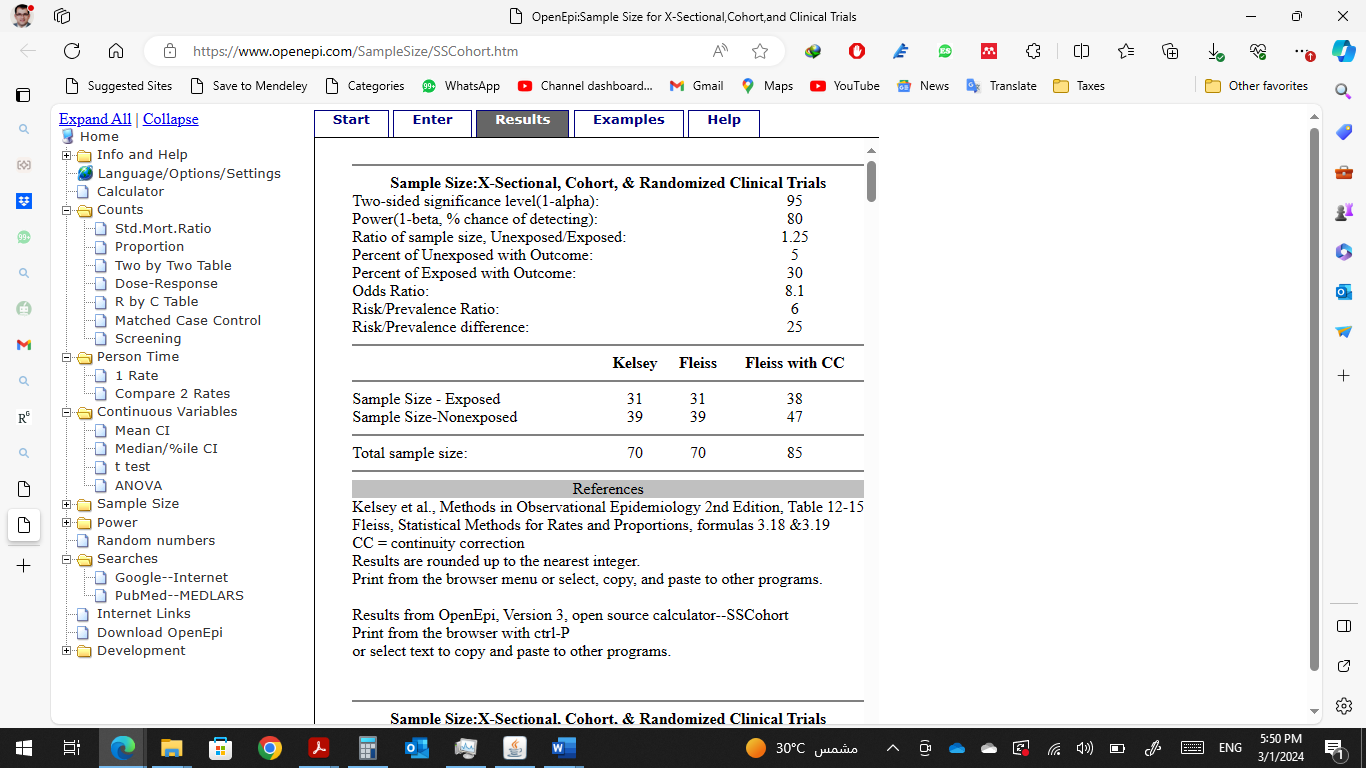


**Supplementary Figure**: showing the calculation of the sample size

Supplement: Supplementary file 1 [file 13018_2024_4994_MOESM1_ESM.docx]
